# Overview of the use of biochar from main cereals to stimulate plant growth

**DOI:** 10.3389/fpls.2022.912264

**Published:** 2022-08-02

**Authors:** Ángela Martínez-Gómez, Jorge Poveda, Carolina Escobar

**Affiliations:** ^1^Facultad de Ciencias Ambientales y Bioquímica, Área de Fisiología Vegetal, Universidad de Castilla-La Mancha, Toledo, Spain; ^2^Institute for Multidisciplinary Research in Applied Biology (IMAB), Universidad Pública de Navarra, Pamplona, Spain; ^3^International Research Organization for Advanced Science and Technology (IROAST), Kumamoto University, Kumamoto, Japan

**Keywords:** biochar, circular economy, main cereal crops, plant growth, recycling

## Abstract

The total global food demand is expected to increase up to 50% between 2010 and 2050; hence, there is a clear need to increase plant productivity with little or no damage to the environment. In this respect, biochar is a carbon-rich material derived from the pyrolysis of organic matter at high temperatures with a limited oxygen supply, with different physicochemical characteristics that depend on the feedstock and pyrolysis conditions. When used as a soil amendment, it has shown many positive environmental effects such as carbon sequestration, reduction of greenhouse gas emissions, and soil improvement. Biochar application has also shown huge benefits when applied to agri-systems, among them, the improvement of plant growth either in optimal conditions or under abiotic or biotic stress. Several mechanisms, such as enhancing the soil microbial diversity and thus increasing soil nutrient-cycling functions, improving soil physicochemical properties, stimulating the microbial colonization, or increasing soil P, K, or N content, have been described to exert these positive effects on plant growth, either alone or in combination with other resources. In addition, it can also improve the plant antioxidant defenses, an evident advantage for plant growth under stress conditions. Although agricultural residues are generated from a wide variety of crops, cereals account for more than half of the world’s harvested area. Yet, in this review, we will focus on biochar obtained from residues of the most common and relevant cereal crops in terms of global production (rice, wheat, maize, and barley) and in their use as recycled residues to stimulate plant growth. The harvesting and processing of these crops generate a vast number and variety of residues that could be locally recycled into valuable products such as biochar, reducing the waste management problem and accomplishing the circular economy premise. However, very scarce literature focused on the use of biochar from a crop to improve its own growth is available. Herein, we present an overview of the literature focused on this topic, compiling most of the studies and discussing the urgent need to deepen into the molecular mechanisms and pathways involved in the beneficial effects of biochar on plant productivity.

## Introduction

The world’s population increases day by day and is expected to reach between 9.4 and 10.1 billion by 2050 (United Nations, Department of Economic and Social Affairs, Population Division, 2019). Similarly, the total global food demand is expected to increase by 35%–56% between 2010 and 2050 (van Dijk et al., 2021), requiring new means of improving agricultural production to ensure the food supply.

Biochar is a carbon-rich material derived from the pyrolysis of organic matter at high temperature with a limited oxygen supply (Lehmann and Joseph, 2009). This process transforms the organic material into variable fractions of solid (biochar), liquid (bio-oil), and gas (syngas) products. The beneficial effect of biochar or charcoal amendments on soil fertility was first described on the black-earth-like anthropogenic amazon soils known as *Terra Preta* (reviewed in Glaser, 2014) derived from soils enriched with black carbon (C) from incompletely burned residues. These soils had enhanced fertility attributed to higher water and nutrient holding capacity, higher pH values, and higher levels of organic matter and nutrients such as potassium (K), calcium (Ca), phosphorous (P), or nitrogen (N; Glaser et al., 2001).

Biochar production depends on the feedstock, heating rate, final temperature, and residence time used, among other parameters. Different thermal treatments can be carried out, including slow and fast pyrolysis, gasification, or torrefaction. However, high-yield and high-quality biochar can be generally obtained with a residence time of a few hours at temperatures around 400°C (Wang D. et al., 2020). Their environmental and health implications need to be analyzed in-depth in order to extend the use of a given biochar, as it can remain in the soil for hundreds of years (Kamali et al., 2022). Some of the problems associated with the use of biochar include the crystallization of organic matter due to high temperatures or the release of heavy metals and aromatic organic compounds, issues that are currently being addressed (Kamali et al., 2022). In this respect, the European Biochar Certificate (EBC) is an international secure control and assessment system that provides guidelines [European Biochar Certificate (EBC), 2012–2022] for sustainable biochar production, processing, and sale, providing customers and producers with a reliable quality standard. It includes limit values for heavy metals and other potential contaminants such as polychlorinated dibenzo-p-dioxins, furans, or polycyclic aromatic hydrocarbons, and it is updated on a regular basis to align with the ongoing development of relevant European legislation and scientific advances. Moreover, according to EBC, the feedstock used for biochar production must be free of non-organic residues, such as plastic or scrap metal, and must be free of organic pollutants, such as paints or solvents (European Biochar Certificate (EBC), (2012–2022)). In addition, and according to the International Biochar Initiative (IBI), the feedstock used should be thoroughly described, specifying for example the composition of the material or whether it was processed or not [International Biochar Initiative (IBI), 2015].

The physicochemical characteristics of biochar are mainly dependent on the feedstock and the pyrolysis conditions used (Yakout, 2017; Chandra and Bhattacharya, 2019). Fixed carbon, recalcitrance, hydrophobicity, aromaticity, pH level, and specific surface area tend to increase with increasing pyrolysis temperature (Enders et al., 2012; Kloss et al., 2012; Angin, 2013; Gai et al., 2014; Jassal et al., 2015; Cheng et al., 2017), while biochar yield, electrical conductivity, cation exchange capacity, and N, hydrogen (H) and oxygen (O) content, decrease (Gai et al., 2014; Cheng et al., 2017).

The application of biochar can benefit the agricultural sector in many ways, as reviewed in Allohverdi et al. (2021). Biochar can improve the stability and water holding capacity of soils (Basso et al., 2013), modify and control microbial soil populations (Bai et al., 2019; Kamau et al., 2019; Chew et al., 2020), and reduce the need for fertilizer as well as fertilizer leaching (Zhang et al., 2021; Zhou et al., 2021). It can also help to ameliorate drought (Yoo et al., 2020), salinity (Liang et al., 2021), and heat stress (Fahad et al., 2016) effects. In addition, it can also protect plants against pathogens (reviewed in Poveda et al., 2021). All these factors should contribute to increased crop production. Aside from agriculture, the incorporation of biochar as a soil amendment has other beneficial environmental effects, e.g., removing inorganic contaminants from soils and water, such as pharmaceuticals (Jung et al., 2015; Du et al., 2021), heavy metals, or pesticides (Cao et al., 2011; Ali et al., 2019); carbon sequestration and reduction of greenhouse gas emissions (Bi et al., 2019; Ginebra et al., 2022) or energy production (reviewed in Bhatia et al., 2021), among others.

Biochar from many different sources has been shown to be effective in improving plant growth or productivity, either from agriculture and forestry residues or from animal/human/farming wastes. Some of the non-agricultural residues used are sewage sludge (Chu et al., 2020), municipal solid waste (Bonanomi et al., 2017), poultry litter (Masud et al., 2020), manure pellet (Pokharel and Chang, 2019), or residues from the brewery industry (Manolikaki and Diamadopoulos, 2020). The forestry residues used are mainly wood (Tartaglia et al., 2020), sawdust (Bonanomi et al., 2020), and paper fiber (Prasad et al., 2018). Although agricultural residues are generated from a wide variety of crops, in this review, we will focus on biochar obtained from residues of the most common and relevant cereal crops in terms of global production (rice, wheat, maize, and barley) and their use as recycled wastes to stimulate plant growth. However, it is important to mention that biochar prepared from other crops, such as peanut (Wu et al., 2019), walnut (Khorram et al., 2018), bamboo (Wang et al., 2019b), cotton (Zhang Z. et al., 2020), sugarcane (Kumar et al., 2021), tomato (Monterumici et al., 2015), coconut (Zhao et al., 2019), soybean (Zheng et al., 2019), etc., are also reported in the literature.

**Figure 1:**
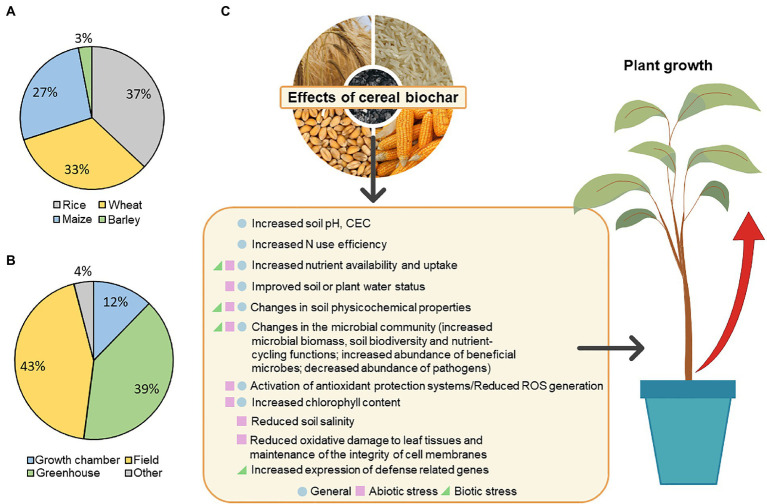
Meta-analyses of publications included in this review and general graphical abstract. **(A)** Percentage of publications on the use of biochar from each cereal (rice, wheat, maize, and barley) to stimulate plant growth. **(B)** Classification of the same publications according to the type of experiments performed with the different biochars. **(C)** Diagram representing the described effects that could influence plant growth after rice, wheat, maize, or barley biochar application.

Diverse mechanisms have been described to promote plant growth, including the increase in nutrient availability and uptake (Zhao et al., 2019; Xi et al., 2020; Cui et al., 2021; You et al., 2021), improved soil physicochemical properties (Huang et al., 2019; Liu et al., 2019), changes in the soil microbial populations (Hansen et al., 2017; Zheng et al., 2018), or changes in gene expression patterns which may influence plant growth (Jaiswal et al., 2020; Mehmood et al., 2020; Figure 1). All these mechanisms will be further explored in the next sections.

Hence, biochar production represents a potential way of transforming residues into valuable products, reducing their waste management problem, and meeting the premise of the circular economy. As mentioned above, in this article, we will focus on the available literature on biochar produced from residues of the main cereal crops (maize, rice, wheat, and barley), and its effect on plant growth promotion, as cereals are the most produced and traded commodity in the world and account for more than half of the world’s harvested area [Food and Agriculture Organization of the United Nations (FAO), 2021a]. This type of feedstock (i.e., residues) is not in competition with land for food or feed production, which could be a relevant factor if the deployment of biochar strategies increases.

## Effects of biochar from major cereal crops on plant growth

The use of biochar to stimulate or improve crop production has a great potential for the development of minimal residue circular economies in which the biochar produced is applied on the same crop that was used as feedstock. For this reason, we reviewed the current available studies of biochar made from residues of cereal crops with a high impact in terms of production within the agri-food system. The main residues used to produce biochar from cereals are husk and straw, which have been reported to stimulate plant growth either in optimal conditions or under abiotic/biotic stress. Herein, we made an overview of the available literature on this topic, compiling all the studies in Table 1 and on a meta-analysis represented in Figures 1A and 1B. It is important to mention that the impact of biochar application on abiotic/biotic stress is not considered as the focus of this review, although the cases where positive effects on plant growth were described are mentioned.

**Table 1:** Compilation of the most relevant studies regarding biochar production from four main cereal crops (rice, wheat, maize, and barley) and their effects on plant growth.

Biochar				Plant system				References
Origin (feedstock)	Application rate	Production method	Combined with	Crop	Growth conditions	Stress	Mechanism of action to stimulate plant growth	
**Rice biochar**								
Rice husk and shell of cotton seed	5% (w/w)	Pyrolysis (400°C)	-	Tomato	Greenhouse, pots	Water stress	Increased soil moisture content	Akhtar et al., 2014
Rice straw	5% (w/w)	Pyrolysis (500°C, 4 h)	-	Rape (*Brassica campestris*)	Greenhouse	-	Increased soil pH, CEC and total C and N. Changes in the microbial community	Xu et al., 2014
Rice husks	2.5% (w/w)	Gasification (900°C–1,100°C)	-	**Rice**	Growth chamber	Heat stress	Improved water status	Fahad et al., 2016
Rice hull	0, 1, 2, and 5% (w/w)	Pyrolysis (500°C)	-	Maize	Growth chamber, pots	Salt stress	Increased stability of water-stable aggregates and P and K soil content	Kim et al., 2016
Rice straw	2.5% (w/w)	Pyrolysis (400°C)	-	Ryegrass	Field	-	Increased soil available P and K	Zhang et al., 2017
Rice husks	20 t ha^−1^	Pyrolysis (600°C, 3 h)	-	**Rice**	Field	-	Increased N uptake and N use efficiency	Huang et al., 2018
Rice straw	3%	Not indicated	Inorganic-phosphate-solubilizing bacteria	Rape (*Brassica napus*)	Field	-	Increased P uptake	Zheng et al., 2018
Rice straw	0, 2.25 and 11.3 Mg ha^−1^	Pyrolysis (500°C, 8 h)	-	**Rice-wheat rotation**	Field, PVC columns	-	Probable release of plant macro- and micronutrients from biochar	Bi et al., 2019
Rice straw	2, 5, 10% (w/w)	Pyrolysis (450°C–550°C)	Dredged sediments	*Phragmites communis*	Sunshine-permeable room	-	Increased N and P uptake. Improved soil water content and photosynthetic rate	Huang et al., 2019
Rice husks	21 g kg^−1^	Pyrolysis (350°C–400°C, 15 min)	*Bacillus pumilus*	**Rice**	Greenhouse	-	Increased soil total C, C/N ratio, exchangeable K^+^, chlorophyll content and nutrient uptake.	Win et al., 2019
Rice husks	1–5% (w/w) in water	Pyrolysis (400°C, 30 min) and liquid extraction with methanol	-	**Rice**	Growth chanber, beakers	-	Increase gene and protein expression of ABP1	Yang et al., 2019
Rice straw	0%, 1%, and 2%	Pyrolysis (400°C, 2 h)	Phosphorus fertilizer	Maize	Greenhouse	-	Increased P availability and soil pH. Decreased soil exchangeable Al^3+^	Baquy et al., 2020
Rice straw	3%	Pyrolysis (450°C, 2 h)	Chitosan	*S*oybean	Growth chamber	Salt stress	Activation of antioxidant protection systems, genetic upregulation, reduced ROS generation and osmolyte development. Increased nutrient uptake and chlorophyll, soluble proteins and sugar contents.	Mehmood et al., 2020
Rice straw	2.8 t ha^−1^	Pyrolysis (500°C, 2 h)	-	**Rice**	Field	-	Changes in the microbial community. Increased total N content and soil available K and Mg	Nan et al., 2020
Rice straw	2% (w/w)	Pyrolysis (400, 800°C)	-	Leaf-used lettuce	Growth chamber	-	Increased soil available N and K, reduced Fe^2+^ and Al^2+^	Xi et al., 2020
Rice straw	7.5 t ha^−1^	Pyrolysis (450°C, 6 h)	Bare urea and controlled-release urea	**Rice**	Field	-	Increase N uptake and N use efficiency	Zheng et al., 2020
Rice straw	15 t ha^−1^	Not indicated	Jasmonic acid	Faba bean	Greenhouse, pots	Salt stress	Reduced oxidative damage to leaf tissues and maintenance of the integrity of cell membranes	El Nahhas et al., 2021
Rice straw	0, 30, 60, and 90 kg fed^−1^	Pyrolysis (500°C, 30 min)	-	Faba bean	Field	-	Not indicated.	Essa et al., 2021
Rice husks and maize stalk (1:1)	10 t ha^−1^	Pyrolysis (350°C, 3 h)	Glycine betaine	**Rice**	Field	-	Improved activity of CAT, APX and POX	Hafez et al., 2021a
Rice husks and maize stalk (1:1)	10 t ha^−1^	Pyrolysis (350°C, 3 h)	Vermicompost	Wheat	Field	Salt and water stress	Increased chlorophyll, proline and carotenoid content; increased relative water content and N, P and K uptake; and increased expression of CAT and APX	Hafez et al., 2021b
Rice straw	1.0% (w/w)	Pyrolysis (450°C, 2 h)	N fertilizer	**Rice**	Pots	-	Increased N use efficiency	Liu Z. et al., 2021
Rice husks and maize stalk (1:1)	10 t ha^−1^	Pyrolysis (350°C, 3 h)	(PGPR; *Azotobacter chroococcum SARS 10* and *Pseudomonas koreensis* MG209738)	Maize	Field	Salt stress	Reduced soil salinity and induced photosynthetic pigments and photosynthesis process	Nehela et al., 2021
Rice straw and waste wood	4 t ha^−1^	Pyrolysis (600°C, 90 min)	N-enrichment	**Rice**	Field	-	Increased levels of soil C and N; increased nutrient retention; increased Fe availability	Yin et al., 2021
Rice husks	2.5, 5, and 7.5 t ha^−1^	Modified biochar kiln (350°C)	-	Tomato	Greenhouse, pots	-	Improved soil physicochemical properties	Adebajo et al., 2022
Rice straw	4.25 g kg^−1^	Pyrolysis (500°C, 5 h)	Rice straw, farmyard manure and mineral fertilizer	Zucchini (*Cucurbite pepo* cultivar Jamila F1)	Greenhouse, pots	-	Improved soil characteristics and increased nutrient availability	Farid et al., 2022
Rice hull	1 t ha^−1^	Pyrolysis (500°C)	Plant growth-promoting bacteria (*Bacillus* spp.)	*Radix pseudostellariae*	Field	Biotic stress (replant disease)	Changed rhizosphere soil metabolites and stimulated proliferation of beneficial microorganisms	Wu et al., 2022
Rice husk	0, 5, 20, and 80 g kg^−1^	Pyrolysis (450°C, 3 h)	-	Chinese crab apple (*Malus hupehensis* Rehd)	Field, pots	Biotic stress (*Fusarium solani*, replant disease)	Increased activity of soil enzymes and decreased abundance of *Fusarium solani*	Wang et al., 2019a
Rice husk	3% (w/w)	Pyrolysis (450°C)	Biocontrol agents (*Bacillus subtilis*, *Trichoderma harzianum*)	Tomato	Greenhouse, pots	Biotic stress (*Meloidogyne incognita*)	Increased expression of defense related genes (*PR-1b*, *JERF3*)	Arshad et al., 2021
**Wheat biochar**								
Wheat straw	20–40 t ha^−1^	Pyrolysis (450°C)	-	Maize	Field	-	Increased N uptake	Zhang et al., 2016
Wheat straw	16 t ha^−1^	Gasification (700–750°C)	-	**Rotation: wheat and oilseed rape**	Field	-	Increased the bacteria and protist populations in soil	Hansen et al., 2017
Wheat straw	10% (w/w)	Pyrolysis (550°C)	-	Barley	Growth chamber	-	Increased P uptake	Shepherd et al., 2017
Peanut shell and wheat straw (1:1, v/v)	0.5–10% (v/v)	Pyrolysis (500°C)	-	Different wild species	Field	-	Enhanced soil biodiversity and nutrient-cycling functions	Chen et al., 2018
Wheat straw	5 t ha^−1^	Pyrolysis (600°C, 3 h)	-	Lentil	Field	-	Increased the organic C content and improved other soil physicochemical properties	Khorram et al., 2018
Wheat straw	8%	Pyrolysis (350°C–550°C)	-	Tomato	Greenhouse, pots	Salt stress	Na^+^ ions adsorption, and release of K^+^, Ca^+2^ and Mg^+2^	She et al., 2018
Wheat straw	1 t ha^−1^	Pyrolysis (500–600°C)	-	**Wheat and rice**	Field	-	Improved soil aggregation and fungal community structure	Bai et al., 2019
Wheat straw	2% (w/w)	Pyrolysis (500°C, 4 h)	-	**Wheat**	Greenhouse, pots	-	Reduced herbicide formesan uptake and increased microbial diversity	Meng et al., 2019
Wheat trash	1% (w/w)	Pyrolysis (450–480°C)	-	**Wheat and subterranean clover**	Greenhouse, pots	P deficiency	Stimulated mycorrhizal colonization, leading to increased P uptake	Solaiman et al., 2019
Wheat straw	1% (w/w)	Pyrolysis (350°C, 30 min)	Compost and biogas slurry	Maize	Greenhouse, pots	-	Increased P, K, N and microbial biomass in soil	Abbas et al., 2020
Wheat straw	0.25% (w/w)	Pyrolysis (400°C, 30 min)	-	Rice	Greenhouse, pots	-	Increased root membrane potential resulting in an increased nutrient uptake. Increased microbial soil diversity	Chew et al., 2020
Wheat straw	20–40 t ha^−1^	Pyrolysis (450°C)	Ammonium nitrate (NH_4_NO_3_)	Pecan	Greenhouse	-	Increased N and enzyme activities in soil	Hou et al., 2020
Wheat straw	0,5% (w/w)	Pyrolysis (500°C)	-	Rice	Greenhouse, pots	-	Increased N soil content	Lu et al., 2020
Wheat straw	15 g kg^−1^	Pyrolysis (300°C, 2 h)	-	Maize	Rhizoboxes	-	Fine root proliferation and increased N and P in soil	Song et al., 2020
Wheat straw	5–10 g kg^−1^	Pyrolysis (550°C)	-	Soybean	Greenhouse, pots	Salt and water stress	Increased N soil content	Zhang et al., 2020
Wheat straw	8 t ha^−1^	Not indicated	-	**Rotation: wheat and maize**	Field	-	Increased soil N content and microbial biomass	Hu et al., 2021
Wheat straw	10% (v/v)	Pyrolysis (750°C, 8 h)	-	**Wheat**	Greenhouse	-	Not indicated	Latini et al., 2021
Wheat straw	20 t ha^−1^	Pyrolysis (550–600°C, 4 h)	-	Rice	Field	-	Increased N uptake and N use efficiency	Liu Y. et al., 2021
Mixed softwood and wheat straw	2% (w/w)	Pyrolysis (550°C)	-	Tobacco	Greenhouse, pots	Water stress	Improved soil hydro-physical properties	Liu X. et al., 2021
Wheat straw	2% (w/w)	Pyrolysis (500°C)	-	Tomato	Greenhouse	Biotic stress (*Ralstonia solanacearum*)	Increased N and P uptake	Tian et al., 2021
Wheat straw	2% (w/w)	Pyrolysis (400°C, 30 min)	-	Rice	Greenhouse	-	Increased N, P, K and Fe uptake	Chew et al., 2022
Wheat straw	20 t ha^−1^	Pyrolysis (450°C)	-	Rice	Field	-	Increased N and P uptake	Liu M. et al., 2022
Wheat straw	2% (w/w)	Pyrolysis (550°C)	-	Tobacco	Greenhouse	-	Increased P and K uptake	Liu X. et al., 2022
**Maize biochar**								
Maize stover	-	Pyrolysis (450°C)	*Bacillus mucilaginosus*	-	-	-	Increased K uptake	Liu et al., 2017
Maize cob	1% (w/w)	Pyrolysis (350°C)	-	Quinoa	Greenhouse, pots	Salt and water stress	Improved the plant antioxidant defense machinery and enhanced nutrient uptake	Ramzani et al., 2017
Maize cob and straw	2.5% (w/w)	Pyrolysis (400°C)	-	Ryegrass	Field	-	Increased P and K uptake	Zhang et al., 2017
Maize straw	1%	Pyrolysis (500°C, 2 h)	-	Rice	Field	-	Increased soil P and Fe content by increasing Fe-reducing bacteria and phosphate-solubilizing bacteria	Xu et al., 2019
Maize straw	15.75–31.5 t ha^−1^	Pyrolysis (500°C)	-	**Soybean and maize**	Field	-	Improved soil aggregation and increased SOC content	Jin et al., 2020
Maize straw	9 kg m^−2^	Pyrolysis (500°C, 2 h)	-	Soybean	Field	-	Increased SOC content	Li et al., 2020
Maize stalk	13.3 g/kg	Pyrolysis (400°C, 1.5 h)	-	Pepper	Greenhouse	Biotic stress (*Phytophthora capsici*)	Increased soil organic matter, and N, P and K content	Wang G. et al., 2020
Maize	2–4% (w/w)	Pyrolysis (600°C, 30 min)	-	Licorice	Growth chamber	Salt stress	Increased soil-microbial enzymatic activity and enhanced nutrient uptake	Egamberdieva et al., 2021
Maize straw	20–50 t ha^−1^	Pyrolysis (500–600°C)	-	**Maize**	Field	-	Increased soil moisture and N uptake	Feng et al., 2021
Maize residue	Water extracts (0.01–0.1%)	Pyrolysis (450°C)	-	Rice	Hydroponic culture	-	Contribution of low molecular weight organic acids	Liu M. et al., 2021
Maize straw	5% (v/v)	Pyrolysis (350–500°C, 1 h)	-	**Maize**	Greenhouse	Salt and water stress	Improved antioxidant defense machinery in plant and enhanced nutrient uptake	Ndiate et al., 2021
Maize seeds	2% (w/v)	Pyrolysis (600°C, 30 min)	Bacteria: *Klebsiella* sp. Fungi: *Talaromyces calidicanius* and *T. purpureogenus*	Lettuce	Greenhouse	-	Supply of N, P and IAA from microbial inoculants	Ma et al., 2022
Maize straw	2% (w/v)	Pyrolysis (400°C)	-	**Maize**	Greenhouse	-	Increased N uptake	Xia et al., 2022
**Barley biochar**								
Barley straw	10 t ha^−1^	Pyrolysis (400°C, 1 h)	Inorganic fertilizer	Chinese cabbage	Field	-	Increased N, P and K uptake	Kang et al., 2018
Barley straw	20 t ha^−1^	Pyrolysis (400°C)	Inorganic fertilizer	Rice	Field	-	Improved soil physical properties and increased soil chemical contents	Kang et al., 2019

Details on biochar characteristics as plant and waste origin, application rate, and production method are given in left columns. Crop description, details on growth conditions and mechanisms of action described to stimulate plant growth are given in columns on the right. In bold, those studies where biochar from a crop was used for its own growth.

## Biochar from rice

Rice (*Oryza* spp.) is one of the most important crops in the world and the primary food source for more than half of the world’s population. With 0.8 billion tons, rice accounts for 8% of global production of the primary crops, being the third after sugarcane and maize. Rice production is led by Asia, with 90% of the total global production [Food and Agriculture Organization of the United Nations (FAO), 2021a]. It is widely cultivated in South Asia, China, Thailand, Japan, and Korea and provides up to 76% of the caloric intake of people in Southeast Asia (Fitzgerald et al., 2009).

Rice processing generates different types of solid waste, the main ones being straw, husk, ash, bran, and broken rice. Rice straw accounts for 50% of the dry weight of rice; it is separated from the grain during harvest and is usually left or burned in the open field, wasting what could be a valuable resource for energy generation, ethanol production, animal feed, or stable litter. Rice husk is a protective layer on the grain (around 20% of the grain’s weight). Unlike straw, rice husk is not appropriate for animal feeding, but it can be used for power or ethanol production and as poultry litter (Moraes et al., 2014). Both residues have shown great potential for biochar production, being the two most used rice by-products. The pyrolysis temperature and residency time for biochar production in most studies usually vary from 30 min to 8 h at 400°C–800°C for rice straw and from 15 min to 3 h at 350°C–1100°C for rice husk (see Table 1). The product yield is highly dependent on the type of reaction and the temperature used, among other factors. As one example, for rice residues with reaction temperatures between 300°C and 800°C, biochar yield is around 36%–65%, while syngas and bio-oil yields are 12%–45% and 22%–45%, respectively (Dunnigan et al., 2018; Fermanelli et al., 2020; Su et al., 2020; Bhatnagar et al., 2022).

The application of rice-derived biochar has shown promising results improving plant growth and plant productivity (Table 1; Figure 1). Rice straw pyrolyzed at 400°C and applied at 2.5% (w/w) increased P and K availability and showed a positive correlation between ryegrass biomass increase and available K (Zhang et al., 2017). Similar results were obtained by Xi et al. (2020), where 2% (w/w) rice biochar application increased soil available N and K, resulting in taller lettuce plants, with longer roots, stronger leaves and stems, as well as greater leaf area. Tomato height, weight, number of flowers, and fruit yield were also improved after 7.5 t ha^−1^ rice husk application (Adebajo et al., 2022), and similar results were obtained in faba bean varieties with higher grain yield, fruit protein content, and plant height (Essa et al., 2021). In both cases, the growth improvement may be related to the impact of biochar on the physicochemical characteristics of the soils. Similarly, Huang et al. (2019) proposed that rice straw biochar contributed to the increase of total soil N content, making it more available to *Phragmites communis* and promoting its growth. But rice biochar can also stimulate C and N cycling by changing the microbial community. For example, increased rape shoot biomass (from 2.31 to 4.23 g) after rice biochar application was the result of an improvement of soil chemical conditions (soil pH and cation exchange capacity) and nutrient availability (total C and N), together with changes in the associated microbiota (Xu et al., 2014).

Rice straw or rice husk biochar has also been used in combination with a variety of other resources such as bacteria, inorganic fertilizers, vermicompost, or dredged sediments (Table 1; Figure 1). For example, Zheng et al. (2018) used biochar as an inoculum carrier for inorganic-phosphate-solubilizing bacteria (iPSB), providing a protective environment for the survival and growth of the iPSB community within the biochar pores, which consequently resulted in increased N availability, P uptake, and rape growth promotion. To study the mechanisms behind the increase in soil P availability due to biochar, Baquy et al. (2020) tested rice straw biochar combined with a P fertilizer (100 mg P kg^−1^) in two different soils, resulting in a significant increase of weight, P uptake and P recovery of maize plants in 2% biochar-amended soils. This increase in dry weight was due to an increased P availability, soil pH, and decreased soil exchangeable Al^3+^. The proposed mechanism responsible for these effects was that functional groups on the biochar surface can compete with soil PO4^3−^ to form complexes with Fe and Al, thereby increasing soil P availability and decreasing exchangeable Al^3+^ (and its associated toxicity). Farid et al. (2022) also described the use of co-composted biochar (70% rice straw, 15% farmyard manure, 10% rice straw biochar, and 5% mineral fertilizer) to improve zucchini growth. The co-composted biochar improved plant height, chlorophyll content, and dry weight, which could be the result of changes in soil characteristics and increased nutrient availability. Moreover, mixed application of biochar and fertilizers could be used to accelerate the restoration of ecosystems. In this respect, Huang et al. (2019) used rice straw biochar alone and with dredged sediments, where a combined application of 50% dredged sediments and 5% straw biochar led to a higher *P. communis* growth rate than the control with only biochar or sediments separately. In general, the addition of dredged sediments increased soil organic C (SOC) while biochar preferentially increased soil P. However, the combination of both improved the physicochemical properties of the soil and altered the rhizosphere microbial community abundance; hence, this method was proposed for the improvement of urban river revetment ecosystems.

Rice straw or rice husk biochar has been proved to be effective in fostering plant tolerance to abiotic stress (Table 1; Figure 1). In this respect, Nehela et al. (2021) used a combination of plant growth-promoting rhizobacteria (PGPR; *Azotobacter chroococcum* SARS 10 and *Pseudomonas koreensis* MG209738) and biochar to alleviate the negative effect of salt stress. This combined application led to higher values of several physicochemical parameters such as chlorophyll, carotenoids, soluble sugars, and relative water content, as well as higher nutrient content (K, N, and P) resulting in improved grain and stover yield of maize plants. A significant increase in the plant water status and tomato fruit yield was also observed by Akhtar et al. (2014) under different irrigation regimes after the application of 5% (w/w) biochar made from rice husk and shell of cotton seeds. In line with this, El Nahhas et al. (2021) tested the combined use of rice straw biochar (15 t ha^−1^) and exogenous jasmonic acid to alleviate the effects of salt stress on faba bean plants. The combined treatment enhanced the growth, the number of flowers, and productivity of salt-stressed plants, as well as their water status and photosynthetic pigments. These results were associated with the maintenance of the integrity of cell membranes and the reduction of the oxidative damage of leaf tissues by enhancing catalase (CAT), peroxidase (POX), superoxide dismutase (SOD), and glutathione reductase (GR) activities. In agreement with this, Kim et al. (2016) applied rice husk biochar to reclaimed tidal land soil, which often contains high levels of soluble salts and exchangeable Na. It increased the soil nutrient content and promoted plant growth since maize dry weight was 101% higher under 5% biochar than the control. The decrease in maize salt stress was attributed to a high K content in the biochar that hindered maize sodium (Na) uptake through competition. Interestingly, K content in the maize tissue was 14% higher than the control, and the decrease in Na tissue content influenced the activity of ascorbate peroxidase (*APX*) and *GR*, both genes associated with the amelioration of oxidative stress. A similar upregulation of genes involved in mitigating oxidative stress was found after the application of a mixture of rice biochar and vermicompost to minimize the effects of soil salinity and water stress on wheat plants. This treatment increased chlorophyll, proline and carotenoid content, N, P and K uptake, and relative water content of wheat plants grown under water stress (50%–75% field capacity) in a saline sodic soil. Vermicompost + biochar also increased the expression levels of *CAT* and *APX* genes, decreasing oxidative stress (Hafez et al., 2021b). Chitosan-modified biochar (CMB) was also effective relieving soybean from salt stress (NaCl 40 mM and 80 mM), as the root length of soybean plants was significantly increased (29% and 31%, respectively) after the application of unmodified biochar (UM), and it was further increased when using CMB (56% and 80%, respectively; Mehmood et al., 2020). Different mechanisms were proposed to explain the stimulation of plant growth by CMB, such as increased nutrient uptake and increased chlorophyll, soluble proteins and sugar contents, which are normally reduced under salt stress. In addition, it promoted the upregulation of antioxidant (*APX*, *CAT*, *SOD*, and *POX*) and salt-tolerance genes (*CHS* and *GmSALT3*). Therefore, CMB minimized the effects of salinity enabling plant protection (Mehmood et al., 2020).

Regarding biotic stress, the application of rice hull or rice husk biochar has reported interesting results (Table 1; Figure 1). A combination of rice husk biochar and biocontrol agents (*B. subtilis* and *Trichoderma harzianum*) was able to enhance the biomass of tomato plants and reduce *Meloidogyne incognita* infection by triggering defense-related genes (*PR-1b* and *JERF3*; Arshad et al., 2021). Rice biochar has also been helpful in alleviating the effects of the replanting disease (mainly caused by the accumulation of soil-borne pathogens; Wu et al., 2022). In this respect, an application of 80 g k^−1^ of rice husk biochar resulted in higher root length, surface area, and volume of apple tree seedlings, reducing the negative effect of the apple replant disease, and actively suppressing *Fusarium solani* infection (Wang et al., 2019a). In a similar way, the combination of rice hull biochar and plant growth-promoting rhizobacteria led to increased leaf area and biomass of *Radix pseudostellariae*, stimulated soil beneficial organisms, and suppressed pathogens through the increased production of soil metabolites, thus alleviating the effects of the replanting disease (Wu et al., 2022).

### The use of biochar from rice residues to promote its own growth

On-site rice biochar application to grow rice (Table 1; in bold; Figure 1) is a returning strategy that contributes in some way to the circular economy, and several studies have indicated its positive effect on rice growth and productivity.

#### Influence of rice biochar on soil biological and chemical properties, nutrient availability, and nutrient uptake for rice growth

The continuous application of 20 t ha^−1^ of rice biochar to a rice field resulted in plant growth promotion and an increase of 14%–26% in soil N uptake, 7%–11% in internal N use efficiency, and a 6% in grain yield (Huang et al., 2018). Bi et al. (2019) showed an enhanced yield (up to 35%) in different rice-wheat rotated soils, probably due to the release of plant macronutrients and micronutrients contained in the rice biochar. Interestingly, biochar-extracted liquor [1%–5% (w/w) in water] also promoted plant height and root growth in rice seedlings. The mechanism of action proposed was based on the overexpression of the *ABP1* gene and the accumulation of its protein product. Accordingly, molecular modeling showed a molecule on the biochar surface that was able to interact with the ABP1 protein, although it was not experimentally proven (Yang et al., 2019). ABP1 was formerly believed as an auxin binding protein with main roles in embryogenesis and postembryonic shoot and root development. However, more recent discoveries showed that *abp1*-null mutants were not compromised for auxin signaling or development, questioning the physiological significance of biochar auxin-binding capacity (Gelová et al., 2021). As previously described, rice biochar can also exert its effects on plant growth by modifying the microbial community of the soil (Table 1). In this respect, Nan et al. (2020) reported a clear improvement in soil bacterial cooperative relationships after treatment with rice biochar in a four-year field trial. The complexity of the rhizosphere bacterial community was enhanced, most probably due to an increase in total soil C content, alongside with an increased total N content and soil available K and magnesium (Mg), which increased rice yield up to 14.5%. Rice biochar has also been used in combination with other resources to promote rice growth. For example, Win et al. (2019) combined rice husk biochar with a biofertilizer (*Bacillus pumilus* strain TUAT-1) to study its influence in two rice genotypes. The biochar-only treatment increased soil total C, C/N ratio, exchangeable K^+^, chlorophyll content, and grain yield, and a positive combined effect of biochar and biofertilizer was observed for plant nutrient uptake, although it was genotype-dependent. Zheng et al. (2020) combined 50% bare urea with 50% controlled-release urea and 7.5 t ha^−1^ of rice straw biochar, resulting in an increase of the yield and N uptake of rice by 71.5% and 91.1%, respectively, when compared to the biochar only treatment, and 10.2% and 7.4% when compared to the same treatment with no biochar. In a similar way, Yin et al. (2021) observed an increased rice yield (38%–41%) after the application of N-enriched rice straw and waste wood biochar, due to increased levels of soil C and N contents, as well as iron (Fe) availability. Liu Z. et al. (2021) combined 1.0% (w/w) rice straw biochar with a N-fertilizer, which enhanced the N use efficiency of rice plants and resulted in increased shoot and root biomass (26%–29%) and grain yield (34%), as well as an enhanced soil microbial biomass.

#### Rice biochar to improve rice tolerance to abiotic stresses

Rice biochar has also been used to ameliorate abiotic stress in rice. In one of the very few studies available, 2.5% (w/w) rice husk biochar was combined with a P fertilizer. This combination was more effective to alleviate the effects of high temperature (32°C–35°C vs. a control temperature of 28°C) than biochar or the fertilizer alone. The main reason for this increase was an improvement in the plant water status (Fahad et al., 2016). Moreover, the dual application of rice husk + maize stalk biochar and exogenous glycine betaine significantly enhanced the growth, physiology, productivity, grain quality, and osmotic stress tolerance of rice plants, as well as nutrient uptake and soil properties, probably due to the activation of the enzymatic antioxidant machinery, i.e., improved activity of antioxidant enzymes including CAT, APX, and POX (Hafez et al., 2021a).

## Biochar from wheat

Wheat (*Triticum* spp.) is one of the first domesticated food crops, being for 8,000 years the main food supply for the major civilizations of Europe, West Asia and North Africa. Nowadays, it is still one of the most widely grown cereals worldwide (more than 219 million ha), being a primary source of nutrients for around 40% of the world’s population. Moreover, as a consequence of its agronomic adaptability, simple storage, and easy conversion of grain into flour, its world trade is greater than for all other crops combined (Giraldo et al., 2019; Food and Agriculture Organization of the United Nations (FAO), 2021b).

Wheat cultivation worldwide produces different types of harvest wastes, mainly husks, and straw (Duque-Acevedo et al., 2020). Throughout the 20th century, the main use of these wastes was as animal feed or stable litter, but other alternatives emerged, such as their incorporation into the field as an amendment by burial (Duque-Acevedo et al., 2020). In the 21st century, new lines of research were developed to obtain products derived from their cellulosic biomass, like bioethanol (Duque-Acevedo et al., 2020).

Wheat harvest residues have been used as raw material to obtain biochar, showing interesting benefits after its application in agricultural systems. The main raw material used for biochar production is wheat straw, although its pyrolysis processing varies from 30 min at 350°C to 8 h at 750°C for most studies (see Table 1). For this type of feedstock and reaction temperatures of 300°C–700°C, biochar production is generally around 16%–47%, while syngas and bio-oil yields are 10%–46% and 4%–52%, respectively (Sanna et al., 2011; Fermanelli et al., 2020; Bhatnagar et al., 2022). The application of biochar from wheat straw to crops other than wheat has reported promising results in terms of plant growth and productivity (Table 1; Figure 1). For example, the application of up to 10% (v/v) of wheat straw biochar increased P uptake in barley plants in controlled conditions (Shepherd et al., 2017), and in maize plants grown in rhizoboxes (application rate of 15 g kg^−1^), with an increased shoot biomass and N use efficiency due to a fine root proliferation and an increase in the amount of N and P in soil (Song et al., 2020). Moreover, the application of this type of biochar in greenhouse pots provided a higher grain yield for rice plants by increasing the soil N content (Lu et al., 2020). In the field, the application of wheat straw biochar (5–40 t ha^−1^) promoted the growth of *Lens culinaris* (lentil) by increasing the organic C content and improving other physicochemical properties of the soil (Khorram et al., 2018), and it was also able to increase maize yield by 23.7% by promoting N uptake (Zhang et al., 2016). In addition, the application of biochar from wheat straw (20 t ha^−1^) in rice fields increased yield by 17%, as a consequence of a higher N and P supply, together with an improvement of more than 10% in the N use efficiency (Liu Y. et al., 2021; Liu M. et al., 2022). The application of 2% (w/w) wheat straw biochar to tobacco plants grown in greenhouses enhanced the rhizosphere C and N stocks and P and K availability, but caused a negative effect on the aboveground N-pool (Liu X. et al., 2022). Interestingly, recent reports point to a relevant mechanism that contributes to the increase in rice biomass and N and P uptake, after treatment with wheat straw biochar (micron-size biochar particles). Yet, a more negative electrical potential at the root epidermal cell layer than at the root surface is created, and this difference may have been the driving force for mineral nutrient absorption (Chew et al., 2020, 2022). Nevertheless, the use of biochar in promoting plant growth can also have other side beneficial effects for the environment. For example, the application of biochar from wheat straw and peanut shell (1:1, v/v) allowed the restoration of degraded soils such as landfill soils, by favoring soil biodiversity and nutrient-cycling functions (Chen et al., 2018), therefore improving plant productivity, species richness, and diversity.

Wheat straw biochar can also be used in combination with other resources (Table 1). In maize plants, wheat straw biochar together with compost and biogas slurry, significantly increased plant height, chlorophyll content, water use efficiency, and grain weight, due to an increase in P, K, N and microbial biomass in the soil (Abbas et al., 2020). When applied together with ammonium nitrate, this biochar increased N content and enzyme activities in the soil, improving the height, chlorophyll content, photosynthetic rate, and N, P, Fe, and K accumulation in *Carya illinoinensis* (pecan; Hou et al., 2020).

Under abiotic stress situations, wheat straw biochar promotes tolerance of different crops (Table 1; Figure 1). In this respect, an increased nutrient supply to plants can improve their tolerance against abiotic stresses, e.g., in tomato plants, wheat biochar amendment increased vegetative growth, yield, and quality parameters under saline irrigation, due to the adsorption of Na^+^ ions and the release of K^+^, Ca^+2^ and Mg^+2^ (She et al., 2018). Similarly, the application of wheat straw biochar in soybean plants subjected to salinity and drought increased the N content in the soil, favoring plant growth (Zhang Y. et al., 2020). Another mechanism through which wheat straw biochar can increase plant tolerance to drought is the improvement of soil hydrophysical properties (soil water content, bulk density, and water-holding capacity) reported in tobacco plants (Liu X. et al., 2021).

Regarding biotic stress (Table 1; Figure 1), the use of wheat biochar has also reported interesting results. In tomato, the use of wheat straw biochar reduced the disease incidence of bacterial wilt caused by *Ralstonia solanacearum* by up to 75%. This was due to an increase in the diversity and activity of rhizosphere microorganisms, together with alterations of the rhizosphere organic acid and amino acid composition. In addition, this increased microbial rhizosphere activity led to an increased supply of N and P to the plants, resulting in an increased plant biomass and length (Tian et al., 2021).

### The use of biochar from wheat residues to promote its own growth

The use of wheat straw biochar in wheat crops (Table 1; in bold) is overall an interesting strategy in the context of a minimal-waste circular economy. In this respect, reusing wheat residues in form of biochar can stimulate wheat growth, likely as a result of favoring the microbial diversity of the soil (Meng et al., 2019; Hu et al., 2021). It can also produce a significant increase in shoot and root biomass, probably as a consequence of a higher P uptake due to a stimulation of mycorrhizal root colonization (Solaiman et al., 2019). In the field, improvements have also been reported in the fungal community structure of soils where wheat straw biochar has been applied, due to a better soil aggregation (Bai et al., 2019). Similarly, improvements in bacteria and protist populations, leading to an increase in K available for wheat plants, were also described following wheat biochar application (Hansen et al., 2017). Moreover, biochar can ameliorate the toxic effects of some persistent herbicides such as fomesafen. It is used to control pre- and post-emergent weeds in crop fields such as soybean and peanut. However, it is toxic to some cereals as wheat, usually used as a rotational crop. The use of biochar from wheat reduced the uptake of this herbicide in wheat, therefore decreasing its toxicity (Meng et al., 2019). However, specific studies should be carried out to adjust the herbicide doses in those complex agri-systems.

Interestingly, the agricultural benefits obtained from the use of wheat biochar as an amendment has led to the development of new products compatible with a sustainable agriculture. One of the recent examples is the formulation of nano-biochar particles with wheat straw biochar and different salts of N, P, K, Ca, Fe, Na, chlorine (Cl) and zinc (Zn), obtaining a nano-fertilizer with a high water-retention capacity and prolonged release of nutrients (Khan et al., 2021).

## Biochar from maize

Maize (*Zea mays* L.) is an important source of carbohydrates for human diets in developing countries and for animal feed in the developed world (Ngoune-Tandzi and Mutengwa, 2020). The annual productivity of maize at present is 1.1 billion tons, being 12% of the total world annual crop production (Food and Agriculture Organization of the United Nations (FAO), 2021a). Maize cultivation worldwide produces mainly husks and straw as harvest wastes, which have been historically used as animal feed, stable litter, soil amendment, or cellulosic biomass for ethanol production (Duque-Acevedo et al., 2020).

Maize harvest residues have been used as raw material to obtain biochar with interesting agricultural benefits (Table 1; Figure 1). In this sense, the main raw material used to obtain biochar from maize residues is straw, varying their processing from pyrolysis for 1 h at 350° (Ndiate et al., 2021) to 30 min at 600° (Egamberdieva et al., 2021; see Table 1). The amount of biochar obtained can change depending on the pyrolysis temperature and the type of thermal treatment used. However, for temperatures between 300°C and 684°C, biochar yield is generally 23%–42%, while syngas and bio-oil yields are 39%–50% and 25%–36%, respectively (Zhu et al., 2015; Chen et al., 2016). As a representative example of the actual production of biochar from cereals, in the case of maize, the production of plant biomass residues is around 8 tons per hectare (Bahri et al., 2018). Considering the use of 50% of those residues for biochar production, as the return of some unprocessed residues is important to maintain the soil organic carbon level in the original soil, and also considering that the pyrolysis process would reduce about one-third the original biomass weight, we would obtain between 1 and 1.5 tons of biochar per hectare of maize. However, as mentioned before, exact biochar yield calculations are almost impossible as it depends on many parameters, and they should therefore be locally assessed considering all the different variables involved.

Interestingly, plant growth promotion could be a consequence of bacterial growth in the rhizosphere following the application of maize stover biochar. This is due to an increased nutrient availability due to the nutritional contribution of the biochar and the microhabitats created within its particles (Liu et al., 2017). In this sense, maize straw biochar increased the biomass of rice plants in the field due to an enhanced number of Fe-reducing bacteria and phosphate-solubilizing bacteria in the soil (Xu et al., 2019). In addition, biochar from maize cob or straw was also capable of promoting plant growth in ryegrass fields by increasing P and K uptake in plants, without the identified action of rhizosphere bacteria (Zhang et al., 2017), or in fields of soybean due to increased SOC content (Li et al., 2020). Furthermore, water extracts from maize biochar could act as a bio-stimulator at a low dosage under hydroponic conditions, as low molecular weight organic acids and nanoparticles contained in the biochar can promote root growth (Liu M. et al., 2021).

The combined use of maize biochar with microbial inoculants can also result in growth benefits for different crops. For example, 18% increase in lettuce dry biomass was achieved through the combined application of biochar from maize seeds and the microorganisms *Klebsiella* sp. (bacteria), *Talaromyces calidicanius* and *T. purpureogenus* (fungi), due to a direct supply of N, P and indole-3-acetic acid (IAA) to the plant by the microorganisms (Ma et al., 2022). Under abiotic stresses, such as drought and salinity, the application of biochar from maize has reported significant increases in plant tolerance (Table 1). In quinoa plants, maize cob biochar increased the plant antioxidant machinery, reducing the accumulation of reactive oxygen species (ROS) and increasing nutrient uptake under drought and salinity stress (Ramzani et al., 2017). However, in licorice plants grown with maize biochar in growth chambers, the increase of plant tolerance under salt stress was a consequence of an increased soil microbial enzymatic activity and nutrient supply to the plant (Egamberdieva et al., 2021).

Under biotic stress, the use of maize biochar can also improve crop responses (Table 1; Figure 1). In pepper, the application of biochar from maize stalk reduced the incidence of Phytophthora blight (caused by *Phytophthora capsica*) by up to 50%, due to an increase in the abundance and diversity of biocontrol fungi within the genus *Aspergillus*, *Chaetomium* and *Trichoderma*. In addition, this biochar also improved soil qualities related to plant growth and development by increasing soil organic matter and N, P, and K content (Wang G. et al., 2020).

### The use of biochar from maize residues to promote its own growth

As mentioned for other crops, the use of biochar from maize harvest residues in maize crops (Table 1; in bold) has a great potential for the development of circular economies. In this way, the application of biochar from maize straw in the field caused a significant increase in the productivity of maize for several years, due to increased SOC and improved soil aggregation (Jin et al., 2020). This enhanced maize productivity could be directly related to a promotion of the photosynthetic rate and an increase in the N utilization rate and water holding capacity of the soil (Feng et al., 2021; Xia et al., 2022). The use of biochar from maize can also be a good strategy to increase the tolerance of maize crops under abiotic stress. One of the most recent studies showed that the application of maize straw biochar under salinity and drought conditions increased the biomass of maize plants by more than 60%, as a consequence of an increase in plant antioxidant activity and nutrient supply (Ndiate et al., 2021).

## Biochar from barley

Barley (*Hordeum vulgare*) is one of the most important cereal crops in the world. In 2020, the global barley production was 157 million tons, with 5 million ha cultivated [Food and Agriculture Organization of the United Nations (FAO), 2021a]. Europe accounts for 61.7% of global barley production [Food and Agriculture Organization of the United Nations (FAO), 2021c], being the third most cultivated cereal in the continent. Barley is primarily used for animal feeding and the brewing industry, with a very low percentage used for human food. Barley processing generates different by-products derived from processes such as pearling, milling, or malting. These by-products (straw, pearling by-products, barley middling’s, hulls, fiber, malt sprouts, etc.) are mainly used for animal feed or stable litter, ethanol production, incorporation in pasta, bread formulations, etc. (Papageorgiou and Skendi, 2018).

Barley straw and spent grain are the main by-products from which biochar is produced. The amount of biochar obtained from these feedstocks depends on the type of thermal reaction and parameters used. However, at pyrolysis temperatures of 460°C–540°C, biochar yield is around 15%–22%, while syngas and bio-oil yields are 37%–44% and 39%–48%, respectively (Sanna et al., 2011). Different studies have shown the potential of barley biochar to improve plant growth (Table 1; Figure 1). In this way, application of 10 t ha^−1^ of barley straw biochar produced by pyrolysis at 400°C had significant positive effects on soil physicochemical properties and increased Chinese cabbage yield in a field experiment (Kang et al., 2018). In addition, after a combined application of biochar + fertilizer, the fresh weight of Chinese cabbage was significantly increased by 112% and 28.5% when compared to the control and the biochar-only treatments, respectively. Similarly, several biometric parameters, such as the leaf number, mean width and length of cabbage, as well as the N, P and K uptake were also promoted under biochar + fertilizer treatments compared to the non-treated control and the biochar-only treatment (Kang et al., 2018). In a similar study, Kang et al. (2019) studied the optimal conditions for biochar application, which were identified as an optimal rate of 20 t ha^−1^, 14 days before rice planting. The results of the biochar application, alone or combined with the inorganic fertilizer under those optimal conditions, significantly increased the culm lengths of rice, the number of grains per panicle, the 1,000 grain weight, and rice yield. Yet, the combined application of biochar + fertilizer had greater effects on all measured parameters than the single application of biochar or a fertilizer alone. There was no literature available on the use of biochar from barley residues for its own growth (Table 1).

Considering all the data presented in this review, the application of biochar shows a general common tendency to stimulate plant growth through several mechanisms, such as enhancing the soil microbial diversity (thus increasing the nutrient-cycling functions in the soil), improving the soil physicochemical properties, stimulating mycorrhizal colonization, increasing the P, K or N content of the soil, or improving the antioxidant defenses of the plant (an advantage under salinity, drought or heat stress; Figure 1). However, a deep knowledge of the molecular mechanisms or pathways involved in plant growth promotion stimulated by biochar is still lacking for many crops as rice, wheat, or maize, particularly when recycled for their own growth. In this respect, it is important to mention that recent data of transcriptomic profiles from tomato plants after the application of biochar from greenhouse-grown pepper, primarily indicated the upregulation of genes and pathways associated with defenses and growth, such as jasmonic acid, brassinosteroids, cytokinins, auxins, and flavonoid synthesis (Jaiswal et al., 2020). Similarly, pepper plants in soils amended with bamboo biochar showed an improvement in plant photosynthesis, energy production, enhanced stress signaling pathways, as well as plant defenses, among others. These effects were tightly coordinated with the differential expression of genes and accumulation of metabolites involved in plant–pathogen interactions, photosynthesis, phenylpropanoid biosynthesis, and protein processing in the endoplasmic reticulum (Zhu et al., 2021). Future research should contribute to increasing this mentioned knowledge in cereal crops.

## Conclusion

In conclusion, a total of 67 publications centered on the use of biochar from main cereals to stimulate plant growth were analyzed, of which 37% were from rice, 33% from wheat, 27% from maize and 3% from barley (Figure 1A). The main raw material used to produce biochar was straw (66% of the total number of studies), i.e., 15 publications on rice, 21 on wheat, 6 on maize, and 2 on barley. Biochar from these main cereals has been primarily used in these studies to stimulate the growth of crops other than the ones used for biochar production (70% of all studies). Furthermore, rice accumulates the largest number of studies focused on the use of its residues as biochar to stimulate its own growth (10 out of a total of 25 publications), followed by maize and wheat (Figure 1). However, generally, biochar has been randomly applied to crops other than those used as feedstock for biochar production. There is, therefore, a general lack of information about the effects of using biochar produced from the same crop. Regarding the type of experiments carried out (Figure 1B), the majority of them were conducted in the field (43%) or greenhouses (39%), a relevant sign of the potential extrapolation of these results for their future application in established agricultural systems.

Hence, biochar is a precious product that can be used as soil amendment with many positive environmental effects such as carbon sequestration, reduction of greenhouse gas emissions, soil improvement, or plant growth promotion. However, in-depth scientific research is still needed in order to be able to apply agricultural cereal residues transformed in biochar locally, which would be compatible with a circular economy. Furthermore, research on how biochar produced from cereal crops can be used to improve the growth of that same crop is still very scarce (in bold, Table 1).

## Author contributions

ÁM-G and JP gathered most of the literature regarding the topic and participated in the writing of the manuscript and the classification of the selected papers in an informative table and a figure (Table 1; Figure 1). ÁM-G made a detailed selection of the papers to focus on the topic. CE participated in the writing, supervised the manuscript structure, selected the papers, and added the critical view. All authors contributed to the article and approved the submitted version.

## Funding

This work was supported by the Spanish Government (PID2019-105924RB-I00 MCIN/AEI/10.13039/501100011033 and RED2018-102407-T) and the Castilla-La Mancha Government (SBPLY/17/180501/000287 and SBPLY/21/ 180501/000033) to CE. The laboratory received support from UCLM intramural funds, and ÁM-G was recipient of a PhD grant from Fundación Tatiana Pérez de Guzmán el Bueno. EU FEDER funds complemented all the grants.

## Conflict of interest

The authors declare that the research was conducted in the absence of any commercial or financial relationships that could be construed as a potential conflict of interest.

## Publisher’s note

All claims expressed in this article are solely those of the authors and do not necessarily represent those of their affiliated organizations, or those of the publisher, the editors and the reviewers. Any product that may be evaluated in this article, or claim that may be made by its manufacturer, is not guaranteed or endorsed by the publisher.
